# Modelling and Characterisation of Orthotropic Damage in Aluminium Alloy 2024

**DOI:** 10.3390/ma17174281

**Published:** 2024-08-29

**Authors:** Nenad Djordjevic, Ravindran Sundararajah, Rade Vignjevic, James Campbell, Kevin Hughes

**Affiliations:** 1 Centre for Assessment of Structures and Materials under Extreme Conditions (CASMEC), Department of Mechanical and Aerospace Engineering, Brunel University London, London UB8 3PH, UK; 2 Department of Applied Mechanics, School of Engineering, Cranfield University, Cranfield MK43 0AL, UK; ravisholan@gmail.com

**Keywords:** damage characterisation, quasistatic loading, elastic–plastic constitutive model with damage, finite element model, VUMAT user material subroutine

## Abstract

The aim of the work presented in this paper was development of a thermodynamically consistent constitutive model for orthotopic metals and determination of its parameters based on standard characterisation methods used in the aerospace industry. The model was derived with additive decomposition of the strain tensor and consisted of an elastic part, derived from Helmholtz free energy, Hill’s thermodynamic potential, which controls evolution of plastic deformation, and damage orthotopic potential, which controls evolution of damage in material. Damage effects were incorporated using the continuum damage mechanics approach, with the effective stress and energy equivalence principle. Material characterisation and derivation of model parameters was conducted with standard specimens with a uniform cross-section, although a number of tests with non-uniform cross-sections were also conducted here. The tests were designed to assess the extent of damage in material over a range of plastic deformation values, where displacement was measured locally using digital image correlation. The new model was implemented as a user material subroutine in Abaqus and verified and validated against the experimental results for aerospace-grade aluminium alloy 2024-T3. Verification was conducted in a series of single element tests, designed to separately validate elasticity, plasticity and damage-related parts of the model. Validation at this stage of the development was based on comparison of the numerical results with experimental data obtained in the quasistatic characterisation tests, which illustrated the ability of the modelling approach to predict experimentally observed behaviour. A validated user material subroutine allows for efficient simulation-led design improvements of aluminium components, such as stiffened panels and the other thin-wall structures used in the aerospace industry.

## 1. Introduction

Growing use of finite element method (FEM)-based virtual structural testing requires valid and robust material models that predict material behaviour under the conditions of interest to specific applications. For applications where damage and failure of structural materials is to be predicted, a concept of continuum damage mechanics (CDM) is typically used, where the mechanical damage in solid materials is due to the creation and growth of micro-cracks or micro-voids. As the scale of these imperfections is too small to be individually represented within an engineering analysis of the structures, their influence within the material is homogenised at the continuum level, so the damage effects are averaged over a volume and represented by a set of continuum variables [1]. 

The CDM models require appropriate characterisation of material parameters, since the quality of these parameters directly influences accuracy of the numerical results. A number of different experimental approaches for measuring parameters that allow derivation of damage model parameters have been proposed and investigated (see, for instance, [1,2] and more recently, [3]). If the method for obtaining these parameters from experiments is complex or time consuming, this limits the use of these models.

A number of damage models have been developed (see, for instance, [4,5,6,7]), with a different level of accuracy and predictive capabilities. The advantage of modelling damage using elastic modulus degradation allows for macro-scale damage representation of the micro-mechanics damage effects, where the average degradation of material properties is obtained by using conventional specimen testing methods. A number of methods of implementing orthotropic behaviour have been developed to date, starting from the early works of Hill [8,9], Bobora [10], Khoei [11], De Borst [12], Aretz [13] and Brunig [14]. Chow and Wang [15,16] proposed a generalised anisotropic damage theory of elasticity and extended it to plasticity and evolution of damage in ductile fractures using continuum damage mechanics based on the energy equivalence principle, developed by Sidoroff [17], Cordebois and Sidoroff [18], Ju [19] and Chow [16]. Chow and Wang introduced a modified damage effect tensor M(D) for virgin material stress equations, which can be applied for general structural analysis. This modelling approach was the basis for a number of recently developed models for metals (see, for instance, [20,21]), composites [22,23] and batteries [24].

The objective of the research described in this paper was modelling and characterisation of damage within an aluminium alloy (AA), AA-2024, modelled as an orthotropic material. The material choice was driven by the aerospace applications, and the lack of material parameters for this commercial alloy. A constitutive model formulation for orthotopic damage was directly adopted from [15,16], but the model was derived for 3D FEM formulation and implemented as a user material subroutine in Abaqus. The model derivation was complemented with the methodology for the material model characterisation. The validated and fully characterised model allowed for simulation-led design of the thin-walled structures used in aerospace applications. 

The paper consists of five sections. Following the Introduction, the damage model formulation is described in Section 2. Section 3 describes the experimental characterisation of the AA-2024-T3 material, including digital image correlation (DIC)-based characterisation of the model parameters. The model implementation and performance are presented in Section 4, which is followed with Conclusions outlined in Section 5. 

## 2. Constitutive Model for Orthotopic Damage

The model derived here is based on a generalised anisotropic theory for ductile damage and fracture, developed by Chow and Wang [16]. The constitutive equations use effective stress tensor $\tilde{\sigma }$, where the anisotropic damage formulation is incorporated by using the damage effect tensor M(D) and energy equivalence principle. The damage effect tensor expressed in the principle material coordinate system is diagonal, written in the following form:

(1) $$M\left(D\right)=diag\left[\begin{array}{cccccc} \frac{1}{1-D_{1}} & \frac{1}{1-D_{2}} & \frac{1}{1-D_{3}} & \frac{1}{\sqrt{\left(1-D_{1}\right)\left(1-D_{2}\right)}} & \frac{1}{\sqrt{\left(1-D_{2}\right)\left(1-D_{3}\right)}} & \frac{1}{\sqrt{\left(1-D_{3}\right)\left(1-D_{1}\right)}} \end{array}\right]$$

where $D_{1},D_{2},D_{3}$ are damage variables defined in the principle material directions.

The proposed model was developed in the framework of thermodynamics and consists of elastic, plastic and damage potentials. The elastic part of the model is derived from Helmholtz free energy being the thermodynamic potential. The plastic potential is determined by the Hill’s anisotropic plasticity tensor H [8,9,25] as:

(2) $$f_{pl}\left(\sigma ,D,R\right)={\tilde{\sigma }}_{pl}-\left[R_{0}+R\left(p\right)\right]=0$$

where $R_{0}$ and R(p) are initial strain hardening threshold and increment in strain hardening, respectively. The equivalent stress ${\tilde{\sigma }}_{pl}$ is defined as:

(3) $${\tilde{\sigma }}_{pl}=\frac{1}{2}{\left[\sigma_{T}:\tilde{H}:\sigma \right]}^{\frac{1}{2}}$$

where Hill’s tensor of damaged material is defined as $\tilde{H}=M_{T}\left(D\right):H:M\left(D\right)$. Evolution of the plastic deformation is described by the monotonically increasing Lagrange multipliers, which are in turn calculated from the consistency conditions (see, for instance, [26]). The expressions for the Lagrange multiplier for plasticity evolution and rate of change of plastic strain (consequently plastic increment) are provided here in the final form without derivation:

(4) $${\dot{\lambda }}_{pl}=\frac{1}{2{\tilde{\sigma }}_{pl}}\frac{\tilde{H}:\sigma :{\tilde{C}}_{e}:\dot{\varepsilon }}{\tilde{H}:\sigma :{\tilde{C}}_{e}:\sigma -\frac{dR}{dp}}$$

(5) $${\dot{\varepsilon }}_{pl}=\frac{1}{2{\tilde{\sigma }}_{pl}}\frac{\tilde{\sigma }-R}{\tilde{H}:\sigma :{\tilde{C}}_{e}:\dot{\varepsilon }-\frac{dR}{dp}}$$

Evolution of the plastic deformation in the current version of the model is controlled by a power law,$R\left(p\right)=Kp_{n}$. 

Damage potential is defined in terms of damage effective stress as:

(6) $$f_{d}\left(\sigma ,D,B\right)={\tilde{\sigma }}_{d}-\left(B_{0}+\left[dB/d\beta \right]\beta \right)=0$$

where ${\tilde{\sigma }}_{d}$ is the equivalent damage effective stress, $B_{0}$ is the initial damage threshold, β is the equivalent damage and dB/dβ is the slope of the damage threshold curve. The equivalent damage effective stress in damage potential is defined as:

(7) $${\tilde{\sigma }}_{d}=\frac{1}{2}{\left[\sigma_{T}:\tilde{J}:\sigma \right]}^{\frac{1}{2}}$$

where the damage characteristic tensor $\tilde{J}$ is defined as $\tilde{J}=M_{T}\left(D\right):J:M\left(D\right)$.

(8) $$\tilde{J}=\left[\begin{array}{cccccc} \frac{1}{{\left(1-D_{1}\right)}^{2}} & \frac{\mu }{\left(1-D_{1}\right)\left(1-D_{2}\right)} & \frac{\mu }{\left(1-D_{1}\right)\left(1-D_{3}\right)} & & & \\ & \frac{1}{{\left(1-D_{2}\right)}^{2}} & \frac{\mu }{\left(1-D_{2}\right)\left(1-D_{3}\right)} & & & \\ & & \frac{1}{{\left(1-D_{3}\right)}^{2}} & & & \\ & & & \frac{2\left(1-\mu \right)}{\left(1-D_{1}\right)\left(1-D_{2}\right)} & & \\ & symm & & & \frac{2\left(1-\mu \right)}{\left(1-D_{2}\right)\left(1-D_{3}\right)} & \\ & & & & & \frac{2\left(1-\mu \right)}{\left(1-D_{1}\right)\left(1-D_{3}\right)} \end{array}\right]$$

The damage characteristic tensor in Equation (8) is symmetric 6 × 6, as suggested by the abbreviation “symm” below the main diagonal; off-diagonal members equal to zero are not presented in the matrix form. Symbol μ denotes the damage potential constant that is defined for orthotropic model formulation in Equation (16) below. 

Similarly to the evolution of the plastic deformation, evolution of the damage variables is determined by the Lagrange multiplier for damage and the consistency conditions, starting from the rate of change of the potential given in Equation (6):

(9) $${\dot{f}}_{d}\left(\sigma ,D,B\right)=\frac{\partial f_{d}}{\partial \sigma }\dot{\sigma }+\frac{\partial f_{d}}{\partial B}\dot{B}=\frac{\partial f_{d}}{\partial \sigma }\dot{\sigma }-{\dot{\lambda }}_{d}\frac{\partial B}{\partial \beta }=0$$

The Lagrange multiplier for damage, and governing equations for the damage variable update, are respectively given as:

(10) $${\dot{\lambda }}_{d}=\frac{\frac{\partial f_{d}}{\partial \sigma }:{\tilde{C}}_{e}:\dot{\varepsilon }-\frac{\partial f_{d}}{\partial \sigma }:{\tilde{C}}_{e}:\frac{\partial f_{pl}}{\partial \sigma }:{\dot{\lambda }}_{pl}}{\frac{dB}{d\beta }}$$

(11) $$\dot{D}=\frac{{\dot{\lambda }}_{d}}{2{\tilde{\sigma }}_{d}}\tilde{J}:\sigma$$

where the damage evolution variable β is updated as:

(12) $$\dot{\beta }=\frac{{\tilde{\sigma }}_{d}-\dot{B}}{\frac{dB}{d\beta }}$$

The material parameters for damage were determined following an approach described in the next section.

## 3. Material Characterisation 

**Figure 1:**
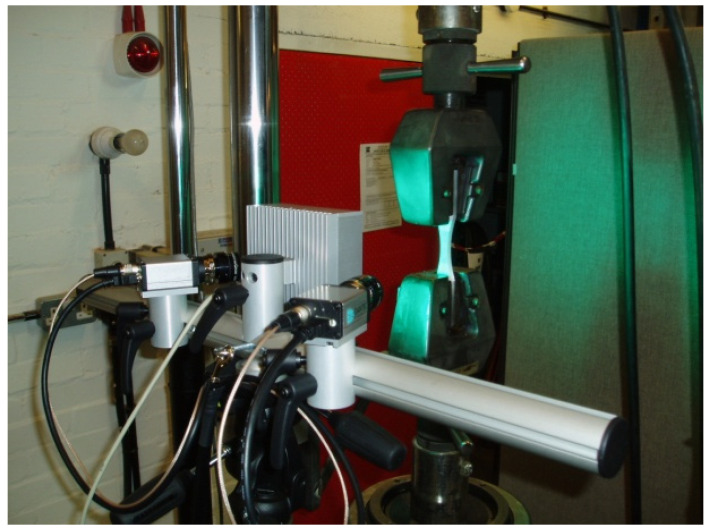
Instron 8032 Servo hydraulic test machine with tensile test specimen and 3D Dantec digital image correlation system Q400.

The uniaxial stress loading–unloading tests were performed with a commercially available aluminium alloy, 2024-T3, which is typically used in aerospace thin-wall structures. The material is typically provided in rolled sheet form, so the tests were conducted with the specimens with rectangular cross sections. Due to this manufacturing process, the material is anisotropic and was tested in three material directions, as described below. The objective of the tests, conducted in a controlled environment, was measurement of the elastic material parameters and the elastic modulus degradation for a range of loading increments up to complete failure. The quasistatic tensile tests were carried out using an Instron 8032 Servo hydraulic machine with a 100 kN load cell and a data logger for displacement measurements. The machine was calibrated with 0.5%, i.e., with the uncertainty of 0.5 KN, which was considered small and was not accounted for in the experimental measurements. The Dantec digital 3D correlation system Q400 was used to obtain the non-contact optical measurement of displacement and strain [27]. The experimental setup is shown in Figure 1. 

**Figure 2:**
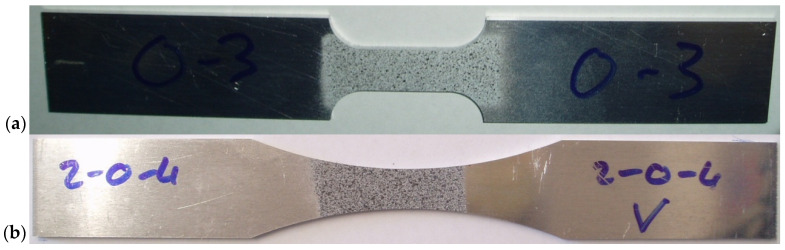
AA2024-T3 specimens used for quasistatic testing: (**a**) standard specimen with uniform size of the gauge cross-section, denoted UCS; (**b**) specimen with non-uniform size of the gauge cross-section, where plastic deformation was localised within a small zone in the middle of the gauge section, denoted VCS.

Two test specimen types shown in Figure 2 were used in the test programme: a standard specimen with uniform cross-sectional size within the gauge section, denoted UCS, and a specimen with non-uniform size of the gauge cross-section, where plastic deformation was localised within the small zone in the middle of the gauge section, denoted VCS. The specimens were cut from a hot-rolled material sheet in three different directions, including the principle material rolling and transverse directions, which were denoted as 0°, 90° and at 45° relative to the rolling directions. It was assumed that material anisotropy originated from a typical rolling process, which may have included solubilisation and/or other treatment processes. It was also assumed that the specimens were manufactured without any residual stress and/or plastic deformation. In total, 22 samples were prepared with uniform cross-sections and non-uniform cross-sections as shown in Table 1. 

The machine-finished specimens were sprayed with speckles of white and black paint in the gauge section, as shown in Figure 2. Specimens were painted with patterns as per the procedure provided by Dantec Dynamics [27]. The paints were free from any erosion or chemical degradation of material. These stochastic patterns formed a reference for the specimen’s initial configuration before any deformation occurred. Displacements were measured using the algorithm provided by Dantec Dynamics. It is based on tracking of the optically recorded patterns, which were calculated relative to the initial reference image and previous deformed positions. 

**Figure 3:**
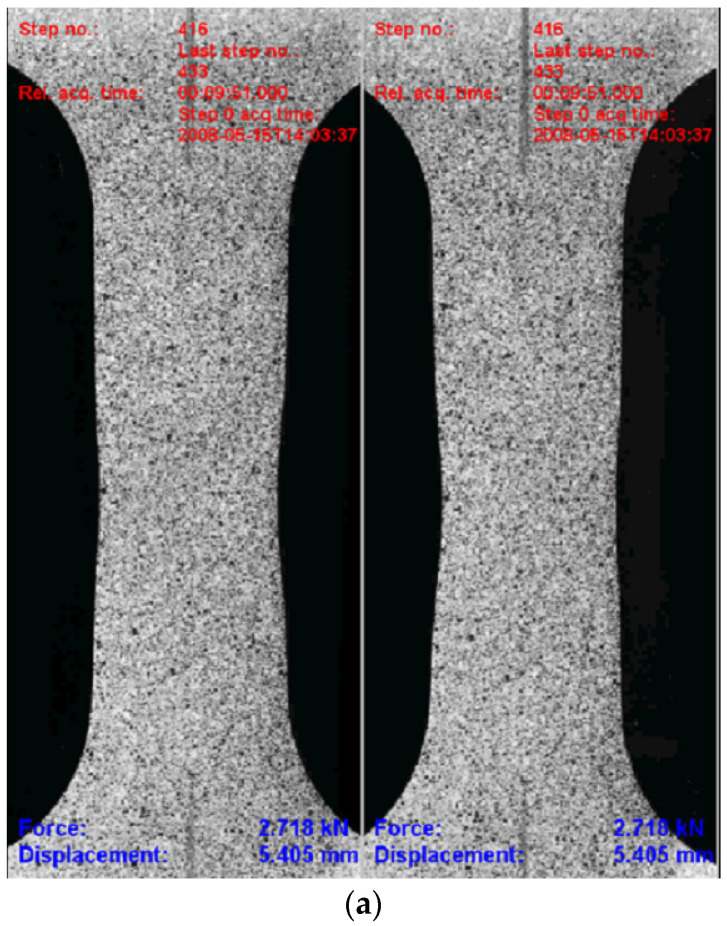

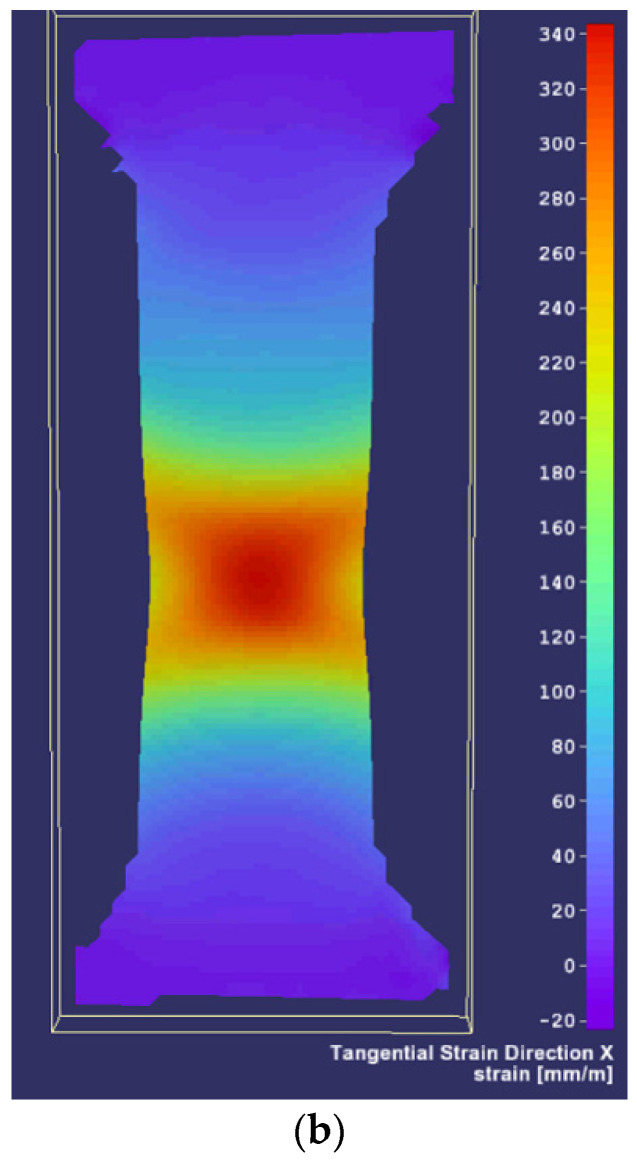
(**a**) UCS specimen sample images of optical measurement using Dantec 3D DIC system Q400 images and (**b**) longitudinal strain surface distribution just before failure (range from 0 to 240).

DIC raw data were processed using the Dantec software ISTRA 4D (software version 4.2.2.15), following one of two procedures: measuring the average surface true strain over the area of interest and/or measuring the displacement of two reference points by placing two pins in the area of interest. The engineering stress was obtained from the force measurements and the initial cross-sectional area of the specimens, which were further used for calculation of the true stress from the true strain. The tensile test specimen and test images obtained during one of the coupon tests are shown in Figure 3 together with the longitudinal strain obtained from the DIC. Figure 3b shows the UCS specimen’s deformed shape with distribution of the longitudinal strain achieved just before the specimen’s complete failure.

**Figure 4:**
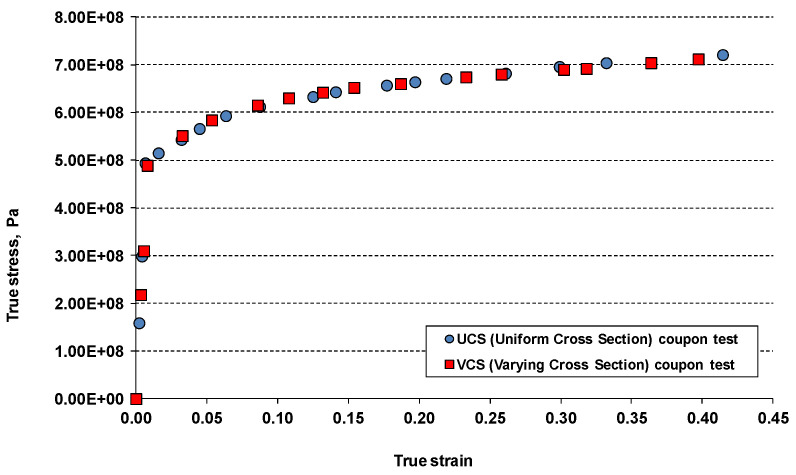
True stress true strain curves obtained with two specimens: standard uniform cross-section (UCS) and specimen with varying cross-section (VSC).

The tests conducted in the same conditions with UCS and VCS specimens revealed an insignificant difference in the true stress, true strain curves obtained with two specimens, as illustrated in Figure 4. Consequently, due to the lower manufacturing cost, the standard UCS specimens were used in the subsequent damage characterisation tests.

### Damage Model Characterisation: Methodology and Results

Material parameters for the damage model were determined experimentally using the following approach. The damage variable in the energy equivalence principle for a one-dimensional problem can be calculated from the current elastic Young’s modulus of damaged material, $\tilde{E}$, and Young’s modulus of virgin material, E as:

(13) $$D=1-\sqrt{\tilde{E}/E}$$

The Young’s modulus of damaged material was determined experimentally for a range of plastic strains from the loading, unloading and reloading tests described below. Having defined the damage variable using Equation (13), one can calculate the effective damaged stress and the equivalent variable for damage evolution β under uniaxial stress loading as:

(14) β=D(1−D/2)

The material parameters $B_{0}$ and B were determined experimentally, with the detailed derivations available in [28]. Making use of Equation (6), the slope of damage threshold is:

(15) $$\frac{dB}{d\beta }=\frac{1}{\beta }\left({\tilde{\sigma }}_{d}-B_{0}\right)$$

The damage potential constant μ can be calculated from the following relationship:

(16) $$\mu =D_{2}\left[1-D_{2}/2\right]/D_{1}\left[1-D_{1}/2\right]$$

**Figure 5:**
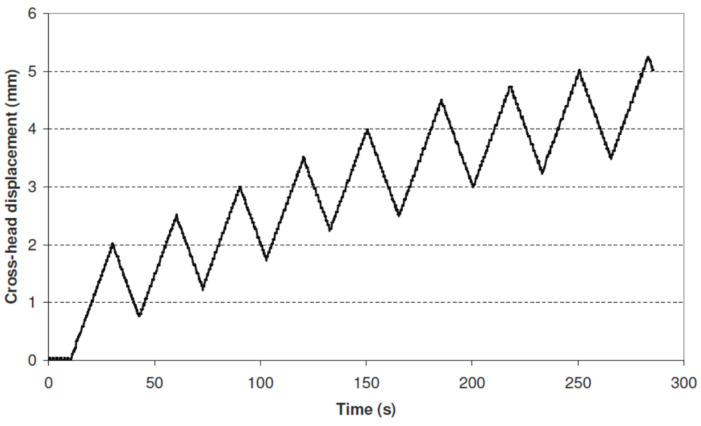
Instron 8032 Servo hydraulic machine cyclic test input (cross-head displacement versus time) used in Dantec Dynamics Q-400 DIC non-contact measurements.

During the uniaxial cyclic test, the material was subject to a repeated loading/unloading cycle, shown in Figure 5, where the maximum plastic strain per cycle was incrementally increased by 2% to 5% until the specimen’s failure. The cross-head displacement in time was obtained for the specified level of plastic deformation during the loading phase, followed by the unloading to zero stress level in each cycle. The signal from the tensile testing machine cross-head displacement against time was used to control the loading–unloading sequence for the cyclic test. This low-cycle test allowed for calculation of degradation of the elastic modulus [3,29], which was then used in Equation (13). 

**Figure 6:**
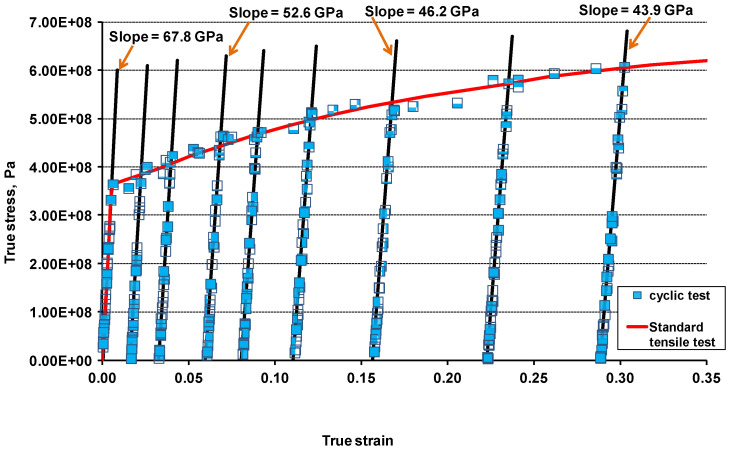
AA2024-T3 cyclic test results in rolling 0° direction with the unloading/reloading slopes that determine elastic modulus degradation due to damage; black lines represent the Young’s moduli of material at a certain level of plastic deformation.

It is well known that forming of micro cracks and micro voids in a material reduce its load-carrying capacity, leading to the deterioration of the Young’s modulus. These reductions in Young’s modulus were calculated from the experimental measurements by using linear regression and are shown in Figure 6 for the longitudinal material direction. 

**Figure 7:**
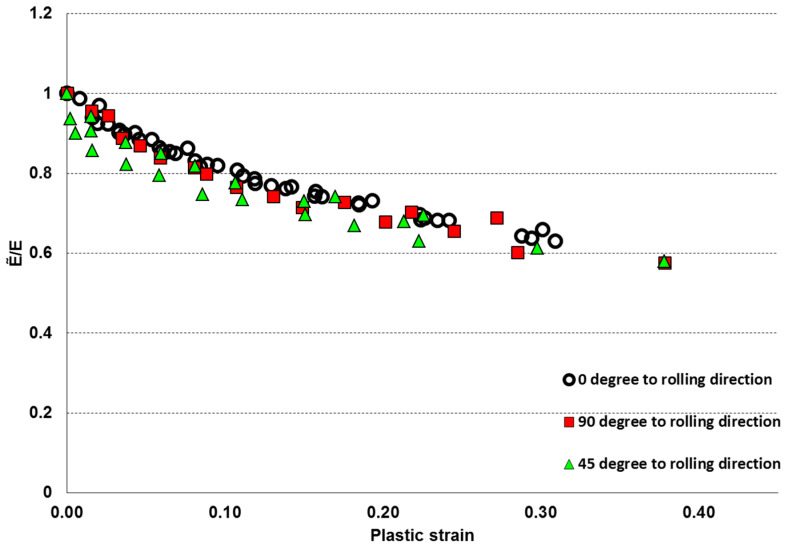
AA2024-T3 uniaxial cyclic test data from coupons for damage characterisation of AA-2024-T3 material on elastic modulus degradation ratio versus plastic strain.

From the cyclic test, the elastic modulus degradation ratios were obtained by dividing the new damaged elastic modulus, $\tilde{E}$, at each cyclic test instance (highlighted with straight line slopes) with the elastic modulus of undamaged/virgin material, E. The plastic strain was obtained from the intersection of the straight line slopes and the strain axis. Damage variables $D_{i}$ were calculated for each loading stage using Equation (13), from zero to maximum value, when the specimen completely failed. Figure 7 shows the complete test results for the AA-2024-T3 material, given in terms of the elastic modulus degradation ratio versus plastic strain. One can note that the material anisotropy is more pronounced at the lower/moderate level of the plastic deformation and deteriorates for the larger plastic strains, so that the graphs obtained for three material directions show only small variation. The orthotropic behaviour might differ based on the grain sizes and distribution of grains, voids, impurities and growth of damage in each material based on the manufacturing process and life cycle loading conditions. The physical and experimental results obtained in this research work reflect the orthotropic behaviour of material recognised by the other researchers. They also confirm that the AA-2024-T3 material showed a low level of orthotropy. 

**Figure 8:**
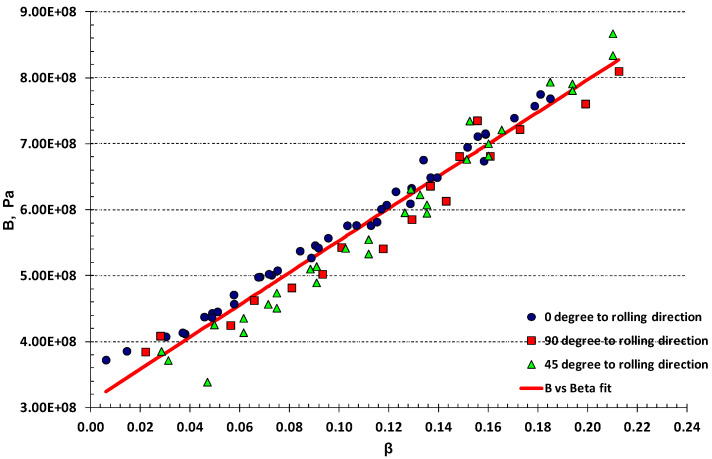
B vs. β fit from uniaxial experimental results of AA-2024-T3 material cyclic tests, R^2^ = 1.

Damage model parameters B and β, calculated from the data and using Equations (13)–(16), are shown in Figure 8, together with linear fitting, where $B_{0}$ intersects with the vertical axis. The results confirm the assumption of linear damage growth, suggested by Chow and Wang [15,16]. 

The critical value of the damage variable, $D_{cr}$, is the maximum allowed amount of damage before complete failure. These critical values are determined for three principle material directions and are denoted as $D_{cr1}$ for the longitudinal (rolling) direction, $D_{2cr}$ for the transversal direction and $D_{3cr}$ for the through-thickness direction. The critical value in the through-thickness direction is not directly measured in this work but was taken equivalent to the transversal direction value, $D_{2cr}$. The orthotropic material parameters are summarised in Table 2.

**Figure 9:**
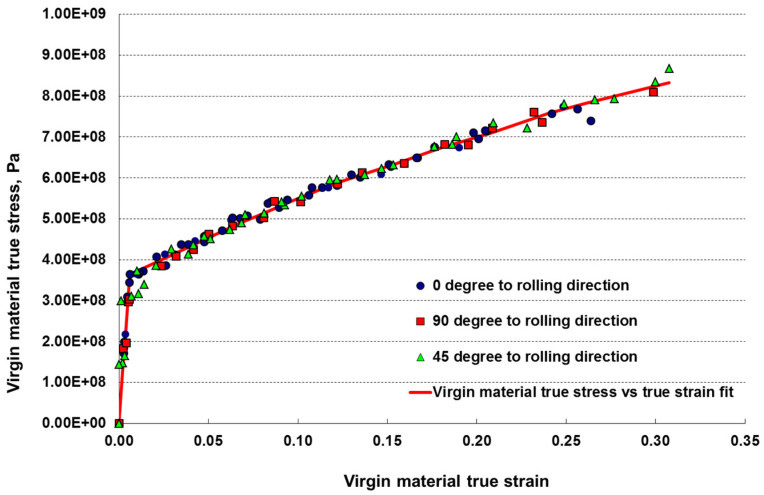
True stress—true strain curves calculated from the uniaxial experimental data. R^2^ = 0.983.

Material constants for the orthotropic material model provided in Table 2 include Hill’s orthotropic coefficients, $R_{11}$, $R_{22}$, $R_{33}$, $R_{44}$, $R_{55}$, $R_{66}$, which are used in Abaqus. These constants were calculated from the original Hill’s coefficients F, G, H, L, M, N using the experimental test results and methodology described in [30]. The material true stress and true strain data obtained from the coupon test are compiled in Figure 9.

The values obtained from these graphs and data from Figure 7, Figure 8 and Figure 9 and Table 2 were used in Abaqus combined with the user material subroutine in simulation of the characterisation tests. Note that the experimentally determined degradation of elastic modulus forms the basis for both the energy equivalence principle and strain equivalence principle. The experimental procedure and damage parameter measurement techniques explained here can be also used for characterisation of the other damage models, which is one of the most significant outcomes of this work.

## 4. Numerical Validation 

**Figure 10:**
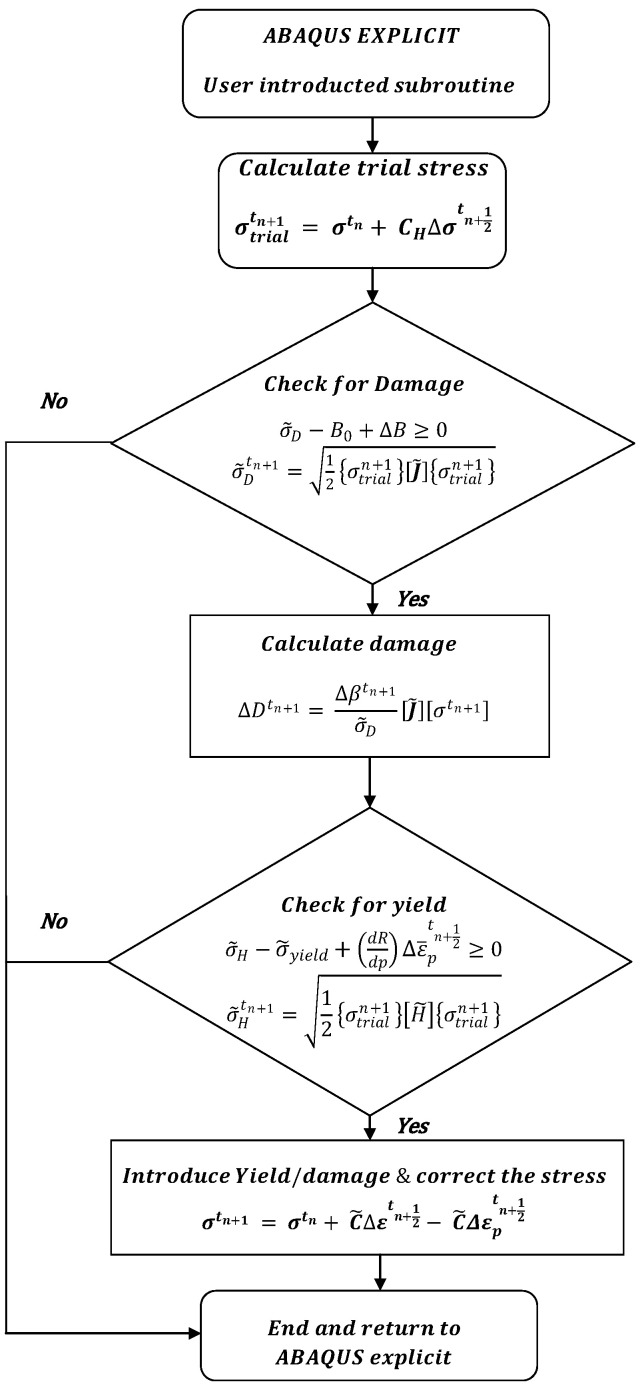
Constitutive model implementation flow chart for the damage model.

The constitutive model was implemented in Abaqus/Explicit as a VUMAT user material subroutine, following the algorithm shown in Figure 10. The present implementation is valid for small deformation problems and small increments of damage growth, where the damage increment in the current step has an insignificant effect on the plastic increment of the current step. 

The Abaqus explicit model calculates trial stress from the total strain increment, which is followed with the plasticity criterion check. If the stress state is outside of the current yield stress, the trial stress is corrected following the normal return procedure (see, for instance, [31]); the new stress is called corrected stress. The corrected stress is then used to calculate the damage variables, which are updated in the iterative steps. Once the damage is calculated, Abaqus explicit updates the stress for the calculated level of damage. 

The model implementation was followed with the verification and validation phase. The former was conducted with a series of single element tests in three stages: linear elastic, elastic with damage, and elastic with plastic and damage. The verification was conducted for both isotropic and orthotropic material types. Validation was also carried out for isotropic and orthotropic material type. 

**Figure 11:**
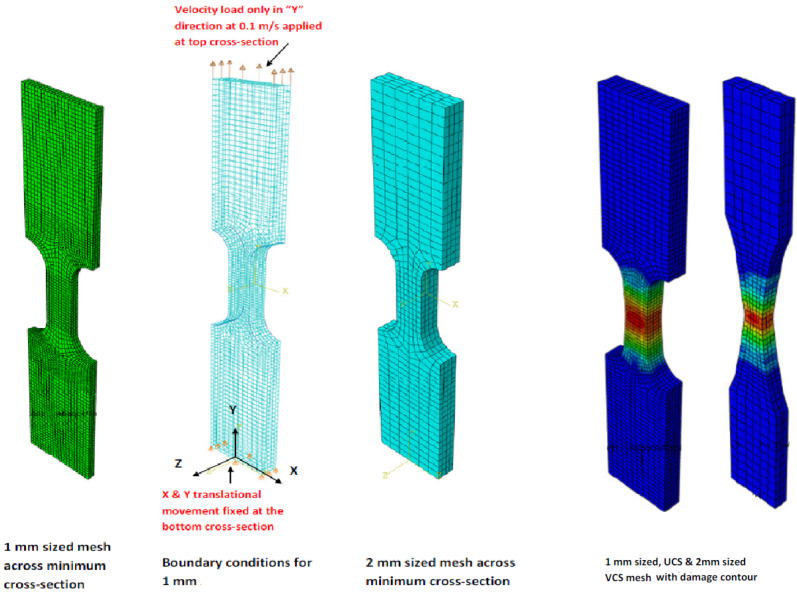
FEM models with considered mesh size, boundary conditions and damage contour output.

The FEM models used for numerical validation against the experimental results are shown in Figure 11. All the models were developed with standard eight-node solid isoparametric elements with reduced integration in Abaqus, denoted as C3D8R. The loading in all simulations was applied on one (top) end as a prescribed velocity of 0.1 m/s. The X and Y constrains were applied at the other (bottom) end of the specimen to achieve uniaxial loading conditions similar to those of the tensile testing. Two mesh densities were considered here: finer with 1 mm edge size in the gauge section, and coarser with 2 mm edge size in the gauge section, which provided equivalent results. 

**Figure 12:**
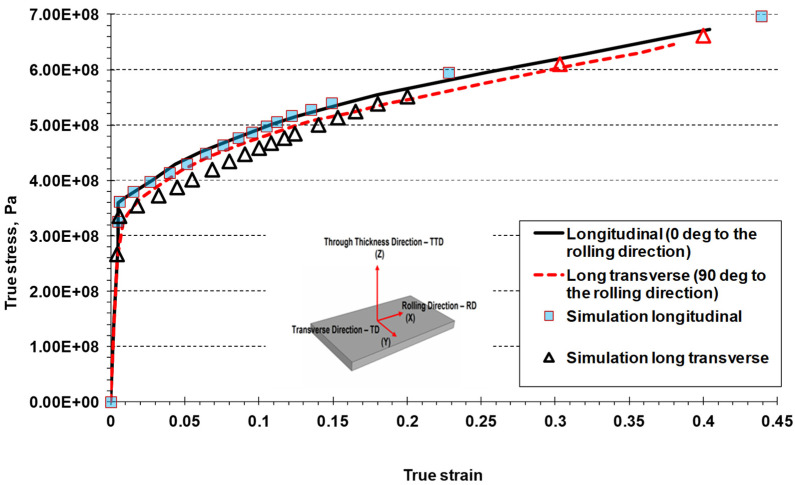
Uniaxial tensile stress test: experimental results versus simulation results.

**Figure 13:**
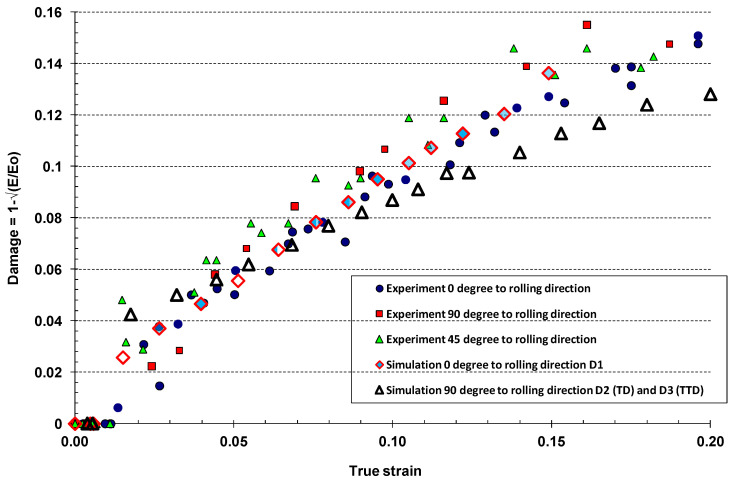
Evolution of damage variables versus true strain: rolling direction, $D_{1}$ (0 degree to rolling direction), transverse direction $D_{2}$ (90 degree to rolling direction or transverse direction (TD)) and $D_{3}$ (through-thickness direction TTD).

Simulation results for the stress–strain curves are compared with experimental results in Figure 12, while comparison of the calculated damage variables is shown in Figure 13. The stress–strain curves, obtained experimentally and in simulation, were equivalent for the longitudinal direction, while discrepancy in the transverse direction was within 5%; the maximum was observed for the lower levels of the plastic strain, for the plastic strain below 10%. 

The damage variables were calculated from uniaxial tests in longitudinal/rolling and transverse directions following the same procedure applied to the experimental results. The simulations produced predictable results for both material directions. The difference in damage parameters in longitudinal direction $D_{1}$ and in transverse direction $D_{2}$ was due to orthotropic properties of the AA2024 material. Figure 13 clearly shows the damage initiation points were not same for the two orthogonal directions, with both the plastic strain and damage initiation starting at lower stresses for the transverse direction compared to the longitudinal direction. The same trend applied to the first phase of the damage and plasticity growth, with damage in the transverse direction being greater than the damage in the longitudinal direction for the same level of plastic strain. This trend changed from the plastic strain of about 15%, where the damage in the transverse direction overcame the level of damage in the longitudinal direction. This may have been due to the smaller material grain size and consequent greater grain density in the transverse direction compared to the rolling direction. Equally, the discrepancy may have originated from the DIC measurement limitations during necking, which constrain the measurement to up to 20% of total strain [27,32]. Nevertheless, the material reached a critical damage level in the transverse direction before the critical damage in the rolling direction in the considered loading scenarios. 

## 5. Conclusions

An orthotropic damage model, coupled with Hill’s orthotropic potential for plastic deformation, was derived in the framework of thermodynamics and implemented in a 3D FEM user material subroutine. A complete methodology for quasistatic material model characterisation was developed and demonstrated in the experimental programme carried out with aerospace-grade AA2024-T3. Material characterisation was based on the damage model parameters calculated from the uniaxial stress cyclic tests, conducted with the standard specimens. It was demonstrated that non-standard specimens with a non-uniform cross-sectional area in the gauge section did not offer any advantage over the standard specimen, which simplifies the sample preparation procedure. Damage parameters calculated using the DIC method were not accurate beyond 20% of the strain due to necking, which is a deficiency of the DIC method. The methodology and experimental data generated in this work can be used with the other damage models and/or characterisation of material behaviour of other ductile alloys. 

The constative model was implemented in Abaqus as a VUMAT user material subroutine and verified and validated in a series of single element tests and characterisation tests. The simulation results were post-processed consistently with the experimental data and produced predictable results. Verification and validation processes and relatively simple damage parameter characterisation using the DIC provide a robust approach for practical applications in the aerospace industry. Rate dependency and thermal softening, and closures of voids, were not included in the model, but there is no constraint for implementation of these features in the future versions of the code.

## Figures and Tables

**Table 1:** AA2024-T3 specimens used for quasistatic testing.

Specimen Orientation Relative to Rolling Direction	UCS	VCS
0°	8	5
90°	1	2
45°	3	3

**Table 2:** AA 2024—T3 material constants for the orthotropic damage model.

Parameter	Description	Experimental Value
$E_{1}$	Young’s modulus—longitudinal or direction-1 or (0°)	67.8 GPa
$E_{2}$	Young’s modulus—transverse or direction-2 or (90°)	66.6 GPa
$E_{3}$	Young’s modulus—through-thickness or direction-3	66.6 GPa
$\nu_{21}$	Poisson ratio between 2-1 direction	0.326
$\nu_{31}$	Poisson ratio between 3-1 direction	0.347
$\nu_{32}$	Poisson ratio between 3-2 direction	0.326
$G_{12}$	Shear modulus between 1-2 direction	25.81 GPa
$G_{23}$	Shear modulus between 2-3 direction	25.81 GPa
$G_{31}$	Shear modulus between 3-1 direction	25.81 GPa
$R_{11}$	Hill’s anisotropic coefficient	1.0
$R_{22}$	Hill’s anisotropic coefficient	0.9364
$R_{33}$	Hill’s anisotropic coefficient	0.8877
$R_{12}$	Hill’s anisotropic coefficient	0.9015
$R_{13}$	Hill’s anisotropic coefficient	0.9515
$R_{23}$	Hill’s anisotropic coefficient	0.9683
μ	Chow’s damage potential matrix constant	0.945
[dB/dβ]	Chow’s damage threshold constant	2.44 GPa
$B_{0}$	Damage initiation stress under uniaxial tensile test	309 MPa
$D_{cr1}$	Critical damage value in longitudinal direction	0.217
$D_{cr2}$	Critical damage value in transverse direction	0.242

## Data Availability

The data presented in this study are available on request from the corresponding author.
